# Digital twins in oncology

**DOI:** 10.1007/s00432-023-04633-1

**Published:** 2023-02-16

**Authors:** Sebastian Sager

**Affiliations:** Department of Mathematics, Otto von Guericke University, Universitätsplatz 2, Magdeburg, 39106 Germany

**What are digital twins to begin with**

Although *twin* is an intrinsically biological phenomenon, so-called *digital twins* made their first appearance in the context of manufacturing and engineering. As is often the case, the exact origins of concept and naming are not commonly agreed upon and depend upon scientific communities. For example, one can easily argue that the underlying concept has been at the core of closed-loop state estimation and control in systems theory many decades before the name *digital twin* became popular. We shall ignore historical context, though, and focus on the author’s subjective point of view on what already has been and what potentially might be transfered to medicine. We shall discuss past and possible (near and far) future usage in oncology, focussing on tumors of the hematopoietic and lymphoid tissues.

The concept of a digital twin usually involves three ingredients: a physical entity in the real world, a digital representation in a virtual world, and information exchange between these two. In our context, the physical entity is certainly a particular human being, often a patient. We start by discussing the two other ingredients in more detail.

 **Digital representation**

Here, *mathematical models* (MM) try to capture the dynamics of biomarkers. Let us first look at an established application of digital twins to get an intuition. Car manufacturers have replaced the expensive and long process of crash-testing physical prototypes by running *simulations* on MMs that capture the most important characteristics of the car. With obvious economic advantages that overcompensate the tedious process of finding a MM. In mechanics, the derivation of a MM that allows to predict the future and answer the question *What would happen if ...?* is possible based on Isaac Newton’s work. Using Newton’s law, acceleration equals force divided by mass, we can write down explicit formulae for the acceleration. Knowing the acceleration and a starting position, we can numerically solve differential equations and hence simulate (predict) the future of the considered isolated system. The main modeling task is to identify the relevant states *x* of the system and all forces acting on them: tedious, but possible.

With a similar approach, it is possible to capture the dynamics of (cancerous) cell counts in a MM. If we denote the number of proliferating cells at a given time *t* as $x_\text {prol}(t)$, the rate of change of this number (similar to velocity or acceleration in the above case) will depend on the value of $x_\text {prol}(t)$. The more proliferating cells there are, the more daughter cells will subsequently increase the cell count. Hence it is a natural first step to assume $\dot{x}_\text {prol}(t) = r \cdot x_\text {prol}(t)$, i.e., a change in time that is proportional to the number of cells with a constant value *r* as the difference between proliferation and cell death. The analytical solution of this equation is a function that is exponential in time *t*, which seems plausible for many situations in population dynamics or disease progression.

From these very basic considerations we can already identify typical challenges in modeling. First, the conceptual relation above involves a *model parameter* *r* which might be different, e.g., depending on patient, mutation of genes, or the time of day. The typical procedure thus involves a fitting of experimental observational data to models, resulting in *personalized model parameters*. How much and what kind of data is necessary depends very much on the MM. Second, the model is using a gross homogenous view on the cancerous cells, ignoring spatial location and particularities (e.g., surrounding tissue for solid tumors or competition for nutrients in the bone marrow) as well as cell cycle, genetic differences between cells, maturity, and so on. Especially for application in radiology or surgery geometric representations of cancerous cell compounds are necessary. Third, the simple MM above is obviously limited to very coarse statements, but can not give any deep insight as to what would happen if, e.g., drugs were administered or how the immune system is affected by the progression of the disease. Deriving MMs that are detailed enough to get answers, but are not over-parameterized has become an art. And it motivates to equip digital twins with multiple MMs of varying levels of detail and characteristics. As a take-away, we note that there is not a unique digital twin, but a collection of different MMs that have to be tailored to a particular purpose (clinical or research question).

 **What is the purpose**

The information exchange comprises the collection of data in real and virtual worlds, running algorithms to create more data, and communicating and applying data. Similar to MMs, the exact mechanisms of interaction and exchange depend on the purpose of the digital twin. We shall have a look at five use cases.

The first use case in oncology is given by a general analysis of the dynamics of biomarkers. MMs without personalization, a simplistic interpretation of a digital twin, can still in some cases be used to derive general treatment rules. E.g., the Norton-Simon hypothesis “Chemotherapy success is proportional to the growth rate of proliferating cancerous cells” and a substitution of the above discussed exponential growth by a so-called Gompertz growth in the MM led to the recommendation of early, dense, high-dosage chemotherapy treatments for breast cancer, a landmark success for mathematical modeling (Simon and Norton 2006; Michor and Beal 2015).

The second use case is the personalization of MMs via longitudinal biomarker data. Algorithms can loop on the steps *measurements*, *state and parameter estimation*, *simulation* and *optimization* of treatments. Results are exchanged between the real and the virtual world. One clinical example is the scheduling and dosage of chemotherapy treatments. Another example is the scheduling of phlebotomies. Such important and complex decisions are usually based on expert knowledge, accumulated throughout the life of a physician and shaped by subjective (and sometimes unconscious) experience. It is not readily transferable and may be unavailable in rural areas. Clinical decision support based on digital twins can help. E.g., a patient suffering from Polycythemia vera might obtain a personalized and optimized phlebotomy schedule based on a MM that captures the individual hematocrit dynamics. With every additional measurement the predicted timings of critical hematocrit values become more accurate, allowing for personalized schedules that try to avoid time intervals in which for whatever reasons (say, a marriage or important business meetings) treatments should be avoided. While a tailored MM (slightly more involved than the one above, consisting of two additional states and a feedback mechanism mimicking EPO) and algorithmic concepts have been published (Lilienthal et al. 2020), a clinical realization has to our knowledge not yet been realized due to ethical, economic, legal, and technical barriers.

A third use case is closest to the digital twin in the original sense of manufacturing and addresses monitoring. Digital twins of cars are able to predict propabilities of fatigue failures of certain parts and give maintenance recommendations before anything bad happens. In the same way, it is perceivable that long-term longitudinal data could be used to monitor changes in biomarkers such as cell counts. It is the subject of current research which biomarkers have a high accuracy in early predictions. One such possibility might be specific metabolites measured via breath gas analysis. A combination with trained machine learning MMs could give predictions of developing cancer, as already shown for other diseases such as major depression (Lueno et al. 2022).

A fourth use case is the training of the next generation of oncologists. In analogy to airplane pilots who have to fly in simulators many miles before they obtain their license, clinical doctors might have to learn cause and effect relations of treatment choices in simulations. While simulated disease progressions certainly lack the intensity of reality, a number of advantages comes to mind: the delay between choice and effect is short and hence better suited for training, a larger number of treatments can be experienced in the same amount of time, the training is also possible in rural areas without many patients, and it is possible to learn from extreme situations that one tries to avoid in real treatments. It is the author’s conviction that learning is most efficient and needed in transient, dangerous situations (in analogy to taking off and landing an airplane).

A fifth use case is a design (or even realization) of clinical studies with cohorts of digital twins. Let us look at the maintenance therapy of acute myeloid leukemia. While it has recently been shown clinically that also here denser chemotherapy treatments have advantages (Jaramillo 2017; Dumas 2020), the huge number of different choices (how to dose and time chemotherapy and G-CSF, how many consolidation cycles with how much delay between them) makes it impossible to design clinical studies for all of them on a trial-and-error basis. However, simulation and optimization studies with digital twins can help to design clinical studies and to find out if a study targeting personalized treatments is worthwhile in the first place. E.g., in Jost (2020) an extended MM combined submodels for myelosuppression, pharmacokinetics and -dynamics of Ara-C and Lenograstim, and the proliferation of leukemic blasts. Optimization showed that in comparison to clinical practice, in 10 out of 13 cases a leukopenia could have been (in simulation) avoided with a modified treatment schedule, while not resulting in increased leukemic blast counts.

 **Perspectives and opportunities**

Looking at the five use cases above, it is not easy to say where the largest potential of digital twins lies. Deriving better clinical protocols from MMs, personalizing treatments for clinical decision support, prevention tools, training of oncologists, or the design of virtual clinical studies may all contribute in their own ways to a better healthcare. Let us focus on perspectives of the last use case, virtual clinical studies. By simulating different individual responses to drug administration, mouse models may be complemented or even completely replaced in the future. In addition to ethical considerations, time-to-clinics can be improved significantly, the whole treatment space (with an almost infinite number of possibilities for how to dose, time, and combine drugs) can be evaluated and the economic costs can be drastically reduced. It is with this in mind that we can understand statements like “...makes it imperative to devise methods of reducing the cost of drug development and one such way is through mathematical modeling” Brady and Enderling (2019), “Consequently, it will remain imperative to use mathematical methods to guide clinical trial design” Simon and Norton (2006), or the suggestive title “Improving Cancer Treatment via Mathematical Modeling: Surmounting the Challenges Is Worth the Effort” Michor and Beal (2015).

 **Current challenges**

Digital twins are at the intersection of many research communities with many active research directions, such as uncertainty quantification, model order reduction, optimal control, numerics, machine learning, measurement technology, or data and knowledge management. In comparison to many engineering applications, the foremost challenges in medical applications seem to be data quality and the difficulty to find tailored MMs. Simplifying, one often differentiates between MMs that are based on domain knowledge (such as the proliferation assumption above or Michaelis-Menten kinetics underlying many pharmacokinetic models) and more general data-driven models such as deep neural networks. The first have the advantages of interpretability, transparency, and a reduced amount of necessary training data. The latter have the main advantage of *universal approximation*, i.e., any functional relation (and not only those that were a priori modeled) can be detected if enough data is available for training. Currently, many research endeavors focus on finding good compromises (hybrid models) that have all of these advantages plus additional properties, such as a reasonable extrapolation quality beyond the training data, intrinsic compliance with scientific laws, or the possibility to interact with other MMs.

 **Recommendations**

Given the enourmous potential of digital twins on so many different levels, the author strongly advocates an interdisciplinary training of the next generation of clinicians and scientists.

Often *personalized (precision) medicine* is associated mainly with genomics. From the author’s perspective it is important to not neglect the exploitation of nonlinear individual dynamics, an inherent feature of digital twins.
